# Ultrasound measurement of umbilical venous flow volume at the intra-abdominal portion in normal fetuses

**DOI:** 10.1007/s10396-023-01315-w

**Published:** 2023-05-12

**Authors:** Katsusuke Ozawa, Seiji Kanazawa, Masashi Mikami, Jin Muromoto, Rika Sugibayashi, Seiji Wada, Haruhiko Sago

**Affiliations:** 1 Division of Fetal Medicine, Center for Maternal-Fetal, Neonatal and Reproductive Medicine, National Center for Child Health and Development, 2-10-1 Okura Setagaya-Ku, Tokyo, 157-8535 Japan; 2 Division of Biostatistics, Clinical Research Center, National Center for Child Health and Development, Tokyo, Japan

**Keywords:** Umbilical venous flow volume, Placental circulation, Umbilical vein

## Abstract

**Purpose:**

Umbilical venous flow volume (UVFV) measured using ultrasound can be used to assess placental circulation in a fetus. UVFV measured at the intra-abdominal portion using half the maximum flow velocity of the umbilical vein (UV) has good reproducibility with low variance. However, reference values in previous reports were based on a small number of cases with a wide reference range. In the present study, we evaluated UVFV standard values measured at the intra-abdominal portion in normal Japanese fetuses.

**Methods:**

Measurements were performed on normal pregnant women during routine ultrasound screening at around 20 or 30 weeks of gestation. The diameter and flow velocity of the UV were measured at the fetal abdomen point between the insertion of the UV and branches of the portal vein. UVFV (ml/min) was calculated as follows: (UV diameter [cm]/2)^2^ × maximum velocity [cm/s] × 0.5 × 3.14 × 60).

**Results:**

A total of 278 pregnant women were included in the study. UVFV increased with gestational weeks, and UVFV per estimated fetal weight (EFW) slightly decreased with increasing gestational weeks. The 50th (10th–90th) percentiles of UVFV per EFW at 20, 25, and 30 weeks of gestation were 130 (105–165), 123 (94–147), and 104 (80–131) ml/min/kg, respectively.

**Conclusion:**

New UVFV reference values measured at the intra-abdominal portion of fetuses using large-scale samples were established. Future studies should assess fetuses under pathologic conditions using UVFV reference values.

## Introduction

Assessing the placental circulation, which reflects the blood supply of oxygen and metabolites to a fetus, is important when examining fetal status. Umbilical venous flow volume (UVFV), which reflects placental circulation, can be measured based on the lumen size and flow velocity in the umbilical vein (UV) using ultrasound. However, UVFV has not yet become a commonly assessed parameter in clinical practice.

One reason for this is that there are various ways to measure UVFV using ultrasound, which leads to large discrepancies among measurements. Regarding measuring sites, the UV at the intra-abdominal portion [1–4] or at the free loop [2, 5–10] is often used. Regarding flow velocity measurement, the Doppler-derived time-averaged flow velocity of the UV and half the maximum flow velocity of the UV are typically used [11, 12]. UVFV measured at the intra-abdominal portion calculated using half the maximum flow velocity of the UV showed a good correlation with the directly measured circuit flow volume in the UV of fetal sheep in the EXTrauterine Environment for Neonatal Development (EXTEND) system [13]. We previously reported that UVFV measured at the intra-abdominal portion using half the maximum flow velocity of the UV showed good reproducibility with low variance [14]. Although there are no other reports regarding the reliability of measuring sites of UVFV, variation of the sites of measurement at the free loop by an examiner might cause large variance in measurements [14].

There have been two reports of UVFV standard values measured at the intra-abdominal portion using half the maximum flow velocity of the UV. However, the first report was based on a small number of cases [2], and the second report had a wide reference range of UVFV distributions [3]. Furthermore, there are no reports of these values in Asians.

In the present study, we evaluated UVFV standard values measured at the intra-abdominal portion in normal Japanese fetuses from the second to the third trimester to promote fetal assessments using UVFV.

## Methods

Pregnant women with singleton pregnancies between 18 and 34 weeks’ gestation who received care at the National Center for Child Health and Development (NCCHD) were recruited between November 2017 and July 2019. Consent for this research was obtained from all participants.

Measurements were performed once during routine ultrasound screening at around 20 or 30 weeks of gestation. Patients with fetal structural or chromosomal abnormalities, small or large for gestational age (estimated fetal weight < − 1.5 or > 1.5 standard deviation [SD]), and maternal complications (e.g., gestational hypertension, gestational diabetes mellitus, and cardiovascular disorder) were excluded. The protocol was approved by the ethics committee of the NCCHD (No. 1644).

We included pregnant women evaluated by two operators to investigate inter-examiner differences [14], as well as those measured by a single operator. Only cases in which UVFV measurements were successfully obtained were included. When UVFV values were measured by two different operators, the average of the two examiners’ measurements was used in the analysis.

Two skilled obstetricians board-certified by the Japan Society of Ultrasonics in Medicine performed transabdominal ultrasound examinations using the Voluson E10 (GE Healthcare, Zipf, Austria) with a convex probe (center frequency of 3.5–6 MHz). The diameter of the UV and the maximum flow velocity (Vmax; cm/s) of the UV were measured according to a previous report [14]. The diameter and flow velocity of the UV were measured at the point of the fetal abdomen between the insertion of the UV and branches of the portal vein. The diameter of the UV was measured from inner wall to inner wall across the lumen of the UV with appropriate magnification of the view with a perpendicular ultrasound beam. The diameter of the UV was calculated as an average of three measurements. The angle between the ultrasound beam and a vessel was kept as close to 0° as possible to measure the velocity of the UV flow, but the angle was allowed to fall within 30° after angle correction. The sample volume of Doppler interrogation was set to be slightly larger than the UV diameter. The maximum velocity of the UV flow was calculated as an average of three measurements. UVFV (ml/min) was calculated as follows: (UV diameter [cm]/2)^2^ × maximum velocity (cm/s) × 0.5 × 3.14 × 60). UVFV per estimated fetal weight (EFW) is also presented, with the EFW calculated using the method recommended by the Japanese Society of Ultrasonography [15].

### Statistical analyses

Scatter plots were created with the number of gestational weeks on the horizontal axis and the mean value of each measurement on the vertical axis. The 5th percentile, 10th percentile, 50th percentile, 90th percentile, and 95th percentile predictions were calculated using the restricted cubic spline regression model and quantile regression and indicated in the scatter plot. Because both ends of the measured gestational weeks were not suitable for calculating the prediction model, the 1% regions at both ends were excluded from the study.

In all studies, the Stata 14.0 (Stata Corp., College Station, TX, USA), R 3.4.1, and SAS 9.4 (SAS Institute, Cary, NC, USA) software programs were used for the statistical analyses.

## Results

A total of 278 pregnant women, including 165 women measured by two operators [14] and 113 women measured by one operator, were included in this study. The characteristics of the pregnant women and fetuses included in this study are shown in Table 1. The first and third quartiles of the gestational duration at ultrasound were 20.4 and 30.3 weeks, respectively. The average ± SD of the gestational age at delivery and the birth weight were 39.1 ± 1.2 weeks and 3021 ± 352 g, respectively.

**Table 1:** Characteristics of pregnant women and fetuses enrolled in the study

Maternal age, years	36 (23, 47)
Primipara	115 (41.5%)
Pregnancy with ART	84 (30.3%)
Height, cm	159.9 ± 5.3
Body weight before pregnancy, kg	52.6 ± 8.4
Smoking	0 (0%)
Gestational week at ultrasound, weeks	24.6 (20.4, 30.3)
Gestational week at delivery, weeks	39.1 ± 1.2
Birth weight, g	3021 ± 352

*ART* assisted reproductive technology

Data are shown as the median (minimum, maximum), average ± standard deviation, or *n* (%)

**Fig. 1:**
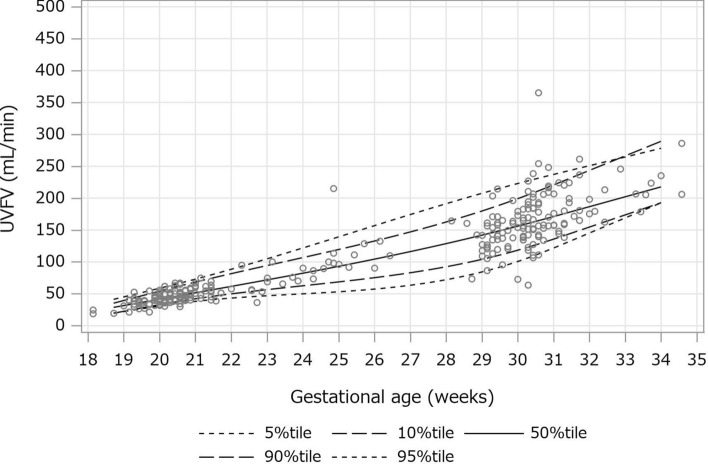
A scatter plot of UVFV between 18 and 34 weeks’ gestation. *UVFV* umbilical venous flow volume

**Fig. 2:**
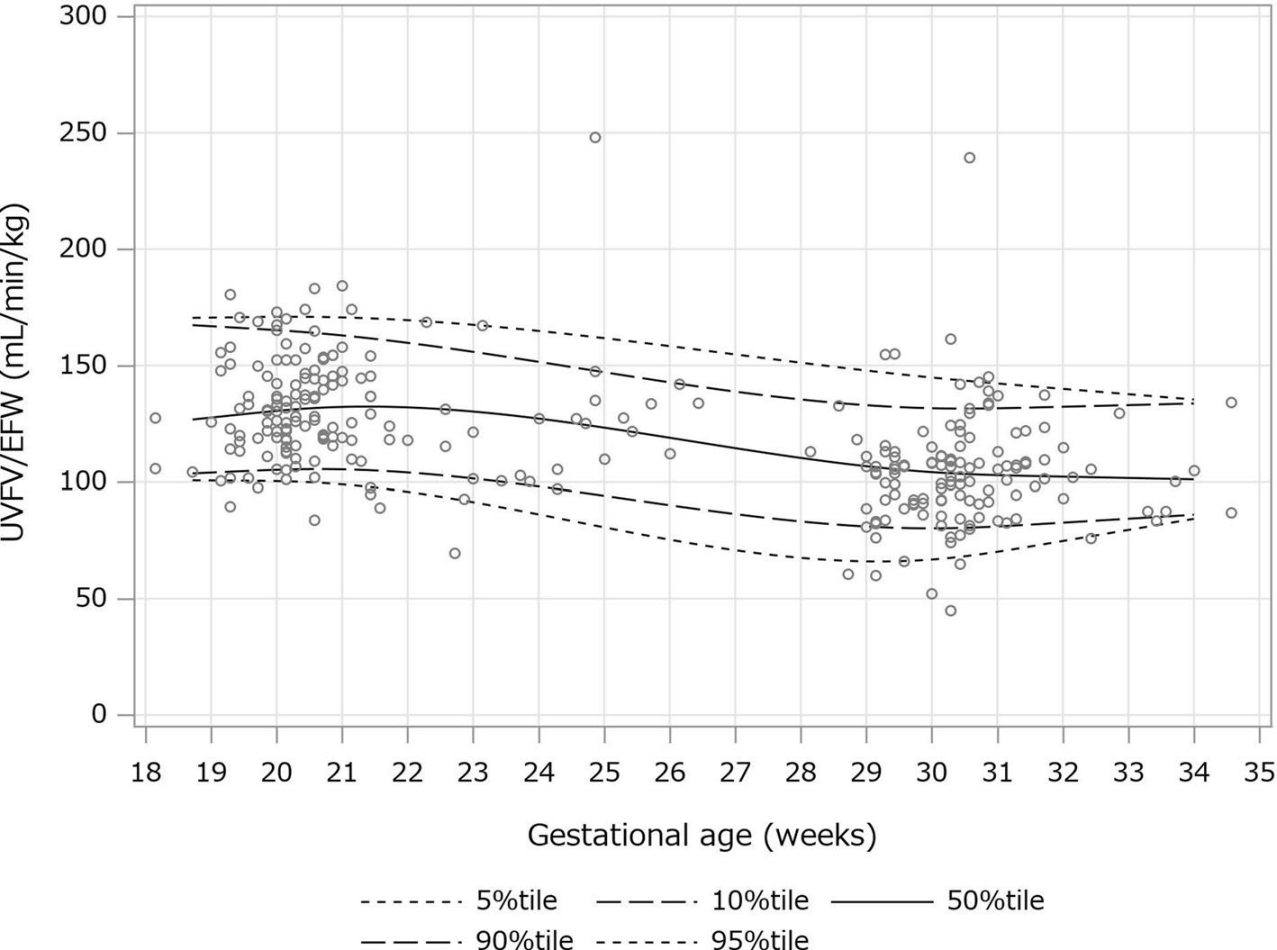
A scatter plot of UVFV per EFW between 18 and 34 weeks’ gestation. *UVFV* umbilical venous flow volume, *EFW* estimated fetal weight

A scatter plot of UVFV between 18 and 34 weeks’ gestation is shown in Fig. 1. UVFV increased with gestational duration. A scatter plot of UVFV per EFW is shown in Fig. 2. UVFV per EFW slightly decreased with gestational duration.

Tables 2 and 3 show the 5th, 10th, 50th, 90th, and 95th percentile of UVFV and UVFV per EFW between 19 and 34 weeks’ gestation, respectively. UVFV increased with gestational duration, while UVFV per EFW slightly decreased with gestational duration. The 50th (10^th^-90^th^) percentiles of UVFV per EFW at 20, 25, and 30 weeks’ gestation were 130 (105–165), 123 (94–147), and 104 (80–131) ml/min/kg, respectively.

**Table 2:** Umbilical venous flow volume (ml/min) between 19 and 34 weeks’ gestation

Weeks	Percentile				
	5%	10%	50%	90%	95%
19	21.8	22.2	31.2	39.0	44.7
20	30.5	32.4	41.1	53.5	58.0
21	38.3	42.3	51.8	68.6	73.3
22	43.3	49.9	61.7	81.6	88.5
23	46.9	56.5	71.7	94.1	104.7
24	49.9	62.4	82.0	106.5	121.4
25	53.1	68.4	92.7	119.0	138.6
26	57.6	75.5	104.7	133.2	157.4
27	63.6	83.2	116.4	147.2	174.5
28	72.1	92.4	128.7	162.6	191.3
29	84.1	103.8	141.8	179.6	207.4
30	100.2	117.9	155.7	198.6	222.5
31	120.8	135.0	170.5	219.8	236.6
32	145.9	155.2	187.0	244.1	251.2
33	169.3	173.9	202.3	266.6	264.6
34	192.6	192.7	217.6	289.1	278.1

**Table 3:** Umbilical venous flow volume per estimated fetal weight (ml/min/kg) between 19 and 34 weeks’ gestation

Weeks	Percentile				
	5%	10%	50%	90%	95%
19	100.7	104.0	127.7	166.9	170.6
20	100.4	105.2	130.5	165.2	170.9
21	99.0	105.4	132.2	162.9	170.7
22	95.8	104.1	132.0	159.7	169.5
23	91.5	101.6	130.3	155.9	167.5
24	86.1	98.0	127.1	151.5	164.8
25	80.7	94.2	123.3	147.1	161.8
26	75.4	90.2	119.0	142.8	158.4
27	70.8	86.3	114.4	138.7	154.7
28	67.6	83.2	110.3	135.4	151.2
29	66.1	81.1	106.8	133.0	147.9
30	66.9	80.2	104.2	131.7	144.8
31	70.2	81.0	102.9	131.6	142.3
32	74.9	82.7	102.3	132.3	139.9
33	79.6	84.4	101.7	133.0	137.7
34	84.3	86.1	101.1	133.7	135.4

## Discussion

The standard values of UVFV measured at the intra-abdominal portion in normal fetuses were established. The number of cases was relatively large and the reference range of the UVFV distributions narrow compared to previous studies. Furthermore, this was the first assessment of UVFV standard values for a Japanese population. UVFV increased with gestational duration, while UVFV per EFW slightly decreased with gestational duration. The 50^th^ percentile of UVFV per EFW at 30 weeks’ gestation was approximately 100 ml/min/kg. The standard values are expected to be useful for fetal assessments using UVFV measurements.

UVFV increased gradually along with gestational duration. This can be explained by the increased cardiac output of a fetus with gestational age, with approximately 25%–30% of cardiac output going through the placental circulation [16, 17]. Previous studies reported results similar to those in our study [2, 3].

Conversely, UVFV per EFW decreased slightly with gestational age. A summary of UVFV per EFW at the intra-abdominal portion of fetuses using half the maximum flow velocity of the UV in this study and two previous reports is shown in Table 4. Bellotti et al. reported that the average UVFV per EFW slightly decreased from 123 ml/min/kg at 20 weeks’ gestation to 109 ml/min/kg at 32 weeks’ gestation [2]. In contrast, Acharya et al. reported that UVFV per EFW slightly increased until 25 weeks’ gestation and then decreased from 117 ml/min/kg at 24 weeks’ gestation to 94 ml/min/kg at 32 weeks’ gestation [3]. Our results were therefore similar to those of Bellotti’s study. Kiserud et al. reported that fetal cardiac output divided by EFW was mostly stable during pregnancy, but the ratio of UVFV to cardiac output decreased with gestational age [17]. These observations may explain the slight decrease in UVFV per EFW with gestational age.

**Table 4:** Published 50th percentiles and 10th–90th percentiles of umbilical venous flow volume (ml/min/kg) measured at the intra-abdominal portion using half the maximum flow velocity of the umbilical vein according to gestational age

Gestational age (weeks)	Present study *n* = 278 Japan	Bellotti et al. *n* = 53 Italy [2]	Acharya et al. *n* = 130 Norway [3]
20	130.5 (105.2–165.2)	122.7	89.4 (50.3–158.6)
24	127.1 (98.0–151.5)	117.3	116.7 (64.7–210.6)
28	110.3 (83.2–135.4)	113.0	110.2 (61.3–198.1)
32	102.3 (82.7–132.3)	109.3	94.1 (52.8–167.4)

It was reported that UVFV was low in cases of fetal growth restriction (FGR) because the distribution of placental blood flow and systemic circulation changes under those conditions [7–9]. In such cases, UVFV decreases, reflecting changes in the placental circulation. Naro et al. reported that UVFV in cases of FGR was lower than that in normal fetuses, but the pulsatility index of umbilical artery flow was not markedly different between these groups [9]. That study further suggested that a quantitative evaluation may allow for assessment of the deteriorating placental circulation earlier than a qualitative evaluation would allow. We therefore should consider assessing any differences in the fetal circulation in monochorionic twin pregnancies by measuring UVFV of both twins. Although some previous studies evaluated UVFV in twin-twin transfusion syndrome [18–20], UVFV can also be assessed in cases of selective FGR. Difference in UVFV between twins may indicate differences in the fetal circulation between twins caused by placental anastomoses among twins or abnormal placental sharing. Therefore, UVFV may be useful for determining the pathology of a fetus. UVFV standard values should aid in evaluating UVFV measurements related to pathological conditions.

Several limitations associated with the present study warrant mention. First, the distribution of gestational weeks for UVFV measurements was biased, with most measurements performed around 20 and 30 weeks’ gestation. Therefore, the restricted cubic spline regression model was used to estimate the edge of the data, which had a small sample size, using linear functions. Second, UVFV standard values were measured in normal fetuses, not the general population. This point should be considered when these standard values are applied. The strength of this study is its large sample size compared to previous studies. In addition, our ranges for the standard values were much narrower than those described in a previous study [3], although further validation would be required.

## Conclusion

In conclusion, we established new reference values for UVFV measured at the intra-abdominal portion of fetuses using a large sample size. UVFV may be a promising tool for evaluating the placental circulation. Further studies are expected to explore assessments of fetuses under pathologic conditions using the UVFV reference values.
